# Neonatal Tracheostomy – Issues and Solutions 

**Published:** 2015-04-01

**Authors:** Saadia A, Prasad GR

**Affiliations:** Department of Pediatric Surgery, Deccan College of Medical Sciences, Hyderabad, India

**Keywords:** Neonate, Tracheostomy

## Abstract

Aims: To record and analyse the technical aspects of neonatal tracheostomy and to suggest some solutions.

Materials and Methods: This is a retrospective observational cohort of 37 cases of neonatal tracheostomies performed over 30 years (1985-2014).

Results: Thirty-three of the 37 tracheostomies were done as an elective procedure and four done emergently. Eighteen neonatal tracheostomies were done with a low transverse cervical incision and 19 were done with low vertical cervical incision. Three patients had bleeding while exposing the trachea. Trachea could not easily be identified in 2 cases. Commercial tracheostomy tubes were used in only 20 cases. In 17 patients, the conventional endotracheal tubes 2.5 or 3fr size were used. There were 3 instances of wound infection out of which one has peri-tracheostomy necrotizing cellulitis and the neonate succumbed to sepsis. Two cases had surgical emphysema. No case had pneumothorax.

Conclusion: We described tracheostomy in neonates in a resource constrained centre. Various makeshift arrangements can be used in absence of standard supplies.

## INTRODUCTION

Tracheostomy has been a known emergency airway access for years but neonatal tracheostomy, the art, the science and the skills are slowly vanishing. Most of the published literature concentrates on complications. The actual technical issue and problems faced are scarcely published. That is why this attempt to analyse a retrospective cohort of 37 neonatal tracheostomies done and to suggest some solutions.

## MATERIALS AND METHODS

This is a retrospective observational cohort of 37 cases of neonatal tracheostomies performed over 30 years (1985-2014). All the records were retrieved from a database managed by senior author in Microsoft access. Four days of mechanical ventilation and neonatologist’s prediction of prolonged ventilation was the common indication. The following parameters have been studied:

A.Indication, 

B.Incision chosen, 

C.Bleeding, 

D.Issues in identifying trachea, 

E.Slippage of trachea, 

F.Slippage of tube, 

G.Dislodged tube, 

H.Difficulties in reintubation, 

I.Tracheal dilator used , 

J.Alternative tube and in absence of commercial endotracheal tube, 

K.Wound infection, 

L.Surgical emphysema, 

M. Too large a wound and other complications. 

The technique followed is similar for planned and emergency cases. The neck is slightly extended. The midline is marked before extending the neck. (this is to avoid inadvertent extension of wound onto chest). In the midline incision all the structures are divided in the line of incision using pen cautery (attaching an intradermal needle to tip of regular cautery will make it a pen cautery). Lowest possible energy is used to cut. The incision is deepened with small, fine, artistic strokes in boluses. Attempt is made to prevent continuous use of diathermy. After exposing the trachea, it is confirmed by palpating tracheal rings (beware neonatal trachea is soft and in laryngomalacia softer). Two thick, 2-0 prolene paramedian full thickness tracheostomy sutures are taken. These sutures are para-median full thickness and the entire team members are informed not to remove them till the decision is taken to take out the tracheotomy. Tracheotomy is done using tip of 11 number surgical blade, neither going onto first ring and nor going too low down. Now, the trachea is dilated either with Hegar dilator or gum elastic esophageal bougie. The tracheostomy tube is made ready. Now the anesthetist is asked to withdraw endotracheal tube very slowly till the tip is seen through tracheotomy and slowly removed to proximal half of tracheotomy. A few sutures are taken approximating the tracheotomy edges to the skin on either side. Once confirmed that tube is in place the anesthetist takes endotracheal tube outside and connects the tracheostomy tube to the ventilator. The tracheostomy tube flanges are again fixed with the tape in flexed neck position and care of the tracheotomy is explained to the entire team. Peri-tracheostomy dressing is done with slit gauze piece. The placement of tracheostomy tube is reconfirmed before leaving operation theatre. A request will be made for chest radiograph to be done at the earliest to exclude pneumothorax.

## RESULTS

Table 1 shows number and incidence of parameters studied.

**Figure:**
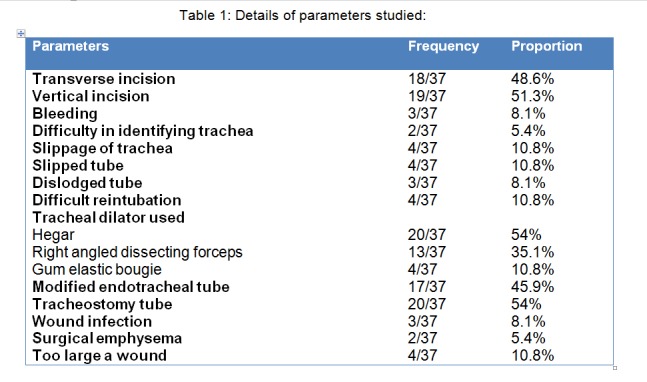
Table 1: Details of parameters studied:

Thirty-three of the 37 tracheostomies were done as an elective procedure for prolonged ventilation. Of the four done as emergency, 2 neonates had massive intraoral tumors presenting at birth, 1 neonate had severe laryngomalacia and 1 had laryngomalacia induced by cervical lymphangiomatosis.

Eighteen neonatal tracheostomies were done with a low transverse cervical incision and 19 were done with low vertical cervical incision. Three patients had bleeding while exposing the trachea; incidentally, all the 3 were transverse incisions.

Trachea could not easily be identified in 2 cases of laryngomalacia.

In 4 patients, the trachea kept on slipping and required an assistants hand to stabilize it before taking stay sutures. Despite tracheal stay sutures the tracheostomy tube went into suprasternal and paratracheal spaces in 4 patients. These 4 also had difficulty in reintubation primarily due to improper placement of stay sutures.

**Figure:**
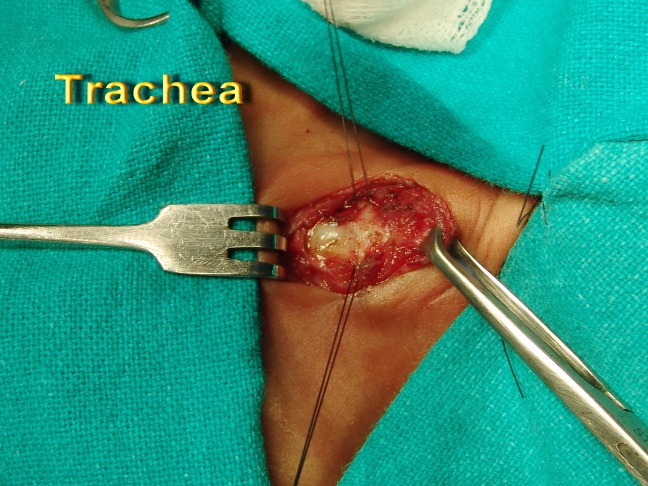
Figure 1: Paramedian stay sutures

Tracheal dilator was not always available. Hence, the senior author (GRP) used Hegar’s dilator in 20 patients, right-angled dissecting forceps in 13 along with Hegar’s and gum elastic bougie in 4 patients.

Despite efforts, commercial tracheostomy tubes were not available at planned time and during emergencies. Only 20 cases commercial tracheostomy tube were available.

In 17 patients, the conventional endotracheal tubes 2.5 or 3 size were cut; 2.5cm length and adaptor was used to connect to the ventilator.

**Figure:**
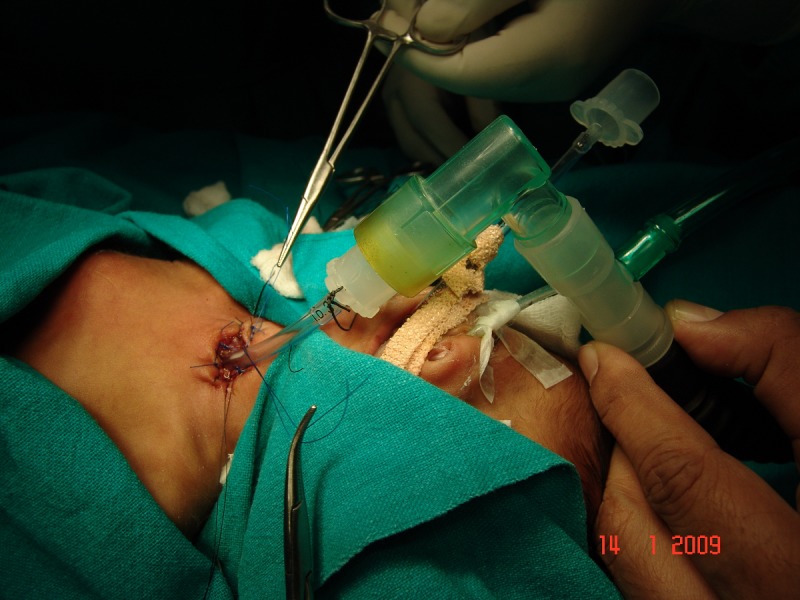
Figure 2: Tracheostomy makeshift tube in situ.

Four patients had too large a wound making suturing trachea to skin difficult.

There were 3 instances of wound infection out of which one has peri-tracheostomy necrotizing cellulitis and the neonate succumbed to sepsis. Two cases had surgical emphysema. No case had pneumothorax.

## DISCUSSION

Neonatal tracheostomy is a common need of newborns requiring prolonged ventilation but the arts and skills of neonatal tracheostomy are progressively vanishing to the extent that a few specialists do neonatal tracheostomy in certain cities. Hence, this was an attempt of analyzing issues and problems and to suggest solutions.

The indications in preterm and term newborns have been well detailed before [1], but details steps of procedure and problems at each step were not published elsewhere. In the present index series, the commonest indication was prolonged mechanical ventilation; 2 cases of massive intraoral tumors and laryngomalacia were other indications for neonatal tracheostomy. Tracheostomy for a massive intraoral tumor has been reported earlier too [2]. 

Increased incidences of morbidity and mortality have been known in preterm babies [1]. In a series of pediatric tracheostomies (n=40), 22.5% early postoperative complications and 35% late complications were noted [3]. In present series, one child died of necrotizing fasciitis around tracheostomy and sepsis.

Though pneumothorax has been known to occur with tracheostomy [1]; the present series did not have one. The authors strongly feel that confining to midline during procedure is the safest way of avoiding pneumothorax.

Other complications like slippage of trachea, inadvertently passage of tracheostomy tube either anteriorly or laterally, inability to reintubate have not been described previously.

Table 1 clearly shows there were 4 cases of slippage of trachea during procedure and 4 cases of slipped tube. In 4 neonates, intubation was difficult as traction sutures were removed inadvertently. Authors stress that the traction sutures are the most vital component of neonatal tracheostomy and these traction sutures should be separately tagged and labeled “not to be removed”.

Other issues like modification of tracheostomy tube have not been dealt with in literatures. Our country due to its special needs and commercial characteristics dedicated neonatal tracheostomy tubes are often unavailable even in planned tracheostomies. The authors had to modify the existing endotracheal tubes upto 2.5x3mm cut short to a length of 2.5-3cm and adaptors are used to connect this to a mechanical ventilator.

Formula to calculate the size of tracheostomy tube on the basis of age and weight is available in standard textbooks. This has helped to be an alternative to commercial neonatal tracheostomy tubes. Although others have stressed upon silicon tracheostomy tubes, only PVC tubes were used in present series. 

Short term complications are mainly bleeding and accidental dislodgement of the tube.

Pereira et al have described a bronchoscopy assisted new technique that restricts dissection strictly to the midline and ensures accurate placement of the tracheostomy below the first tracheal ring [4]. We have no experience of the same.

## Conclusion

The difficulties and issues of neonatal tracheostomy are analysed step by step. Some solutions are suggested based on practical experience on 37 neonatal tracheostomies.

## Footnotes

**Source of Support:** None

**Conflict of Interest:** None

